# Shaped cathodes for the production of ultra-short multi-electron pulses

**DOI:** 10.1063/1.4974779

**Published:** 2017-01-25

**Authors:** Ariel Alcides Petruk, Kostyantyn Pichugin, Germán Sciaini

**Affiliations:** Department of Chemistry and Waterloo Institute for Nanotechnology, University of Waterloo, Waterloo, Ontario N2L 3G1, Canada

## Abstract

An electrostatic electron source design capable of producing sub-20 femtoseconds (rms) multi-electron pulses is presented. The photoelectron gun concept builds upon geometrical electric field enhancement at the cathode surface. Particle tracer simulations indicate the generation of extremely short bunches even beyond 40 cm of propagation. Comparisons with compact electron sources commonly used for femtosecond electron diffraction are made.

## INTRODUCTION

The field of ultrafast structural dynamics is quickly growing, as shorter and brighter hard X-ray and electron pulses are being produced and implemented to light up atoms in motion.^1–8^ The advent of forth generation light sources^9,10^ has made the production of ultra-bright femtosecond (fs) hard X-ray pulses possible, which have been successfully applied for time-resolved diffraction^11–14^ and ultrafast coherent imaging.^15–17^ On the other hand, the use of ultrashort electron bursts has also emerged as a powerful means to atomically resolve dynamical phenomena and structure in the laboratory setting.^18–35^ In this regard, different approaches for the generation of fs multi-electron bunches have been developed to meet the prerequisite time-resolution to observe the movement of atoms; i.e., sub-picosecond electron pulses and ideally the shorter the better to avoid temporal blurring in stroboscopically recorded images. Compact femtosecond electron diffraction (FED) instruments with electrostatic electron guns, based on quasi-flat cathode and anode electrodes, have enabled a time-resolution of ≅100 fs (rms, root-mean-square deviation) with bright multi-electron pulses.^36,37^ For simplicity, electron pulses were assumed to be Gaussian in shape, and therefore a conversion factor of 2.355 has been used to calculate fwhm (full-width-at-half-maximum) from rms values. Recent designs with a minimal cathode-to-sample distance have brought the temporal resolution of these sources closer to the limit imposed by their initial energy spread—or single electron pulse limit.^30,38–41^ Electron kinetic energies (*KE*) produced by electrostatic guns typically range from sub 1 keV to 100 keV and are commonly referred to as sub-relativistic. More advanced electron sources based on radio frequency (RF) photo-injectors are known to generate ultrashort bright pulses of relativistic electrons (*KE* > 1 MeV). This technology is relatively mature within the accelerator community due to its use in synchrotron and free electron laser facilities and has become popular for monitoring ultrafast structural dynamics.^42–49^ As its energy spread gets under control, laser-driven electron acceleration is also arising as a low-cost alternative for the generation of ultrashort and ultrabright electron pulses with *KE* in the 200 keV–1 GeV range.^50–53^ In addition, different active and passive electron pulse compression schemes have been proposed and/or demonstrated.^27–29,53–64^ One of the most successfully applied methods in recent FED experiments with ultrabright electron bursts relies on the use of an RF (or microwave) cavity that acts as a temporal lens.^27–29,55,56,60–63^ This methodology was found to compress dense sub-relativistic multi-electron pulses down to 67 fs (rms).^60^ Shorter multi-electron pulses are expected from this approach for which synchronization noise has limited the instrument response to about 80–150 fs (rms).^60–62^ However, a recent phase-lock scheme based on passive optical enhancement has reduced this timing jitter to only ≅5 fs (rms).^65–67^ Therefore, RF pill-lens electron pulse rebunching still holds great promise in providing sub-20 fs (rms) temporal resolution with bright multi-electron bunches.^56^ Furthermore, all-optical electron pulse compression throughout the use of a single cycle THz resonator has recently shown to bring the duration of multi-electron pulses from 395 fs (rms) to 32 fs (rms) [930 fs (fwhm) to 75 fs (fwhm)] with minimal long-term timing drift ≅ 4 fs (rms).^68,69^ This method is expected to generate even shorter multi-electron bursts.^68^

## RESULTS AND DISCUSSION

**FIG. 1.**
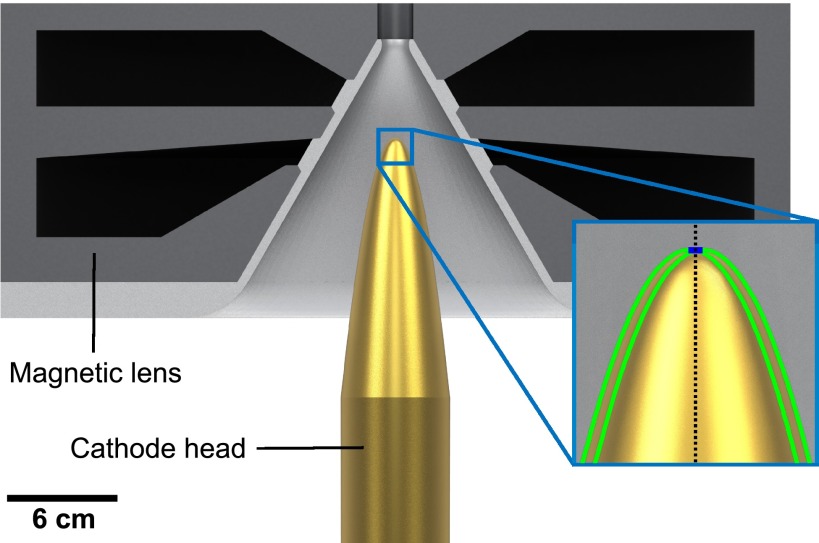
CAD of the electron source concept. The main components are a parabolically shaped photocathode head with a small flat region and a double magnetic lens system with a conical inner form. This cathode shape has been carefully selected in order to confer an on-axis geometrical surface electric field magnitude of 50 MV/m without exceeding a maximum of 60 MV/m in other parts of the head. The in-lens system ensures *B_z_* = 0 at the electron birth to avoid magnetic emittance growth. The conical anode shape helps to maintain the surface electric field at the anode $\left|E_{a}\right|≲5\;\text{MV}/m$ and brings the magnetic poles closer to the cathode head. The magnetic lens system has been optimized in order to obtain, within practical constraints, a reasonable electron spot size at the sample position and low power consumption to avoid water-cooling. Inlet: black dotted line corresponds to the symmetry *z*-axis; green curves depict two parabolas displaced from the propagation axis by 0.5 mm in the radial direction and which follow the equation $f\left(r\right)=1.6\;\;{\text{cm}}^{-1}\;r^{2}$; blue segment highlights the flat region of 1 mm in diameter.

Here, we introduce a rather simple all-electrostatic electron gun design that delivers multi-electron bursts as short as 12 fs (rms) [28 fs (fwhm)] at a relatively long electron propagation distance of 10 cm (sample position in our instrument) without the need of electron pulse temporal rebunching. A 300 kV FED setup based on this source concept is under construction at the University of Waterloo. The electron gun exploits the advantage of strong on-axis electric field acceleration at the electron birth. Figure 1 shows a computer-aided design (CAD) of the key electron source components alongside a geometrical depiction of the photocathode head. The cathode surface has a parabolic shape with a small flat circular area of 1 mm in diameter centered at the symmetry axis or electron propagation axis defined as  (0, 0, z). This flat region is necessary to avoid an excessive kick in the transverse direction acting on off-axis electrons that greatly deteriorates the transverse and longitudinal properties of the electron bunch. The cathode is positioned inside a double magnetic lens (in-lens system). The magnetic fields generated by each lens point in the opposite direction along the *z*-axis in order to provide a resultant field *B_z_* = 0 at the cathode surface (0, 0, 0). This is a necessary condition to null emittance growth caused by magnetic fields at the electron pulse birth.^70^ This in-lens source design yields a lateral spot size ≅ 190 *μ*m (rms) for a quasi-parallel electron beam at the sample's plane. A normalized transverse emittance of 0.025 mm mrad (or transverse coherence length of about 3 nm) was obtained. This value suffices for the study of most inorganic and organic crystalline materials composed of small molecules. The lens system has been optimized to operate under the assumption of a core material with a relative magnetic permeability of 10^4^ and a saturation magnetic field of 1.5 T. Such values are easily attainable by various soft magnetic iron alloys.^71^ The required total power was estimated to be only 200 W.

Local electric field enhancement by several orders of magnitude (≅1 GV/m) is a well-known effect in field emitters and single electron to a few electrons photo-triggered tip sources.^72–78^ Recently, such nanoemitters have been successfully applied to monitor photocurrents in nanostructures^77^ as well as ultrafast structural dynamics.^78^ The introduced cathode head exploits the use of moderate geometrical field enhancement while permitting the generation of multi-electron bunches.

**FIG. 2.**
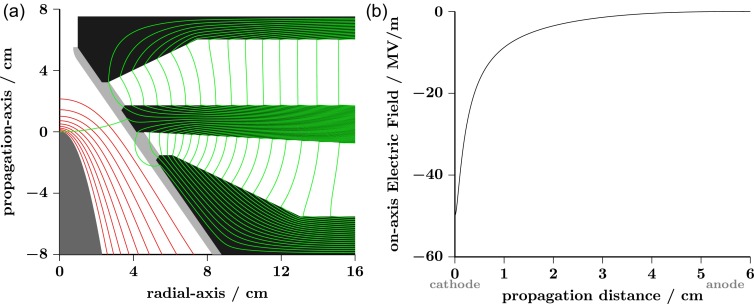
(a) Schematic of the electron source concept. Equipotential and magnetic field lines are shown in red and green, respectively. (b) Electric field values obtained along the centrosymmetric axis (0, 0, *z*) for a cathode held at −300 kV. Calculations were done using Poisson Superfish.^82^

Electrons in simulations were generated at the cathode surface considering a temporal (longitudinal) Gaussian profile of 6 fs (rms) [14 fs (fwhm)], an initial energy spread of 0.2 eV, and a lateral (transverse) Gaussian spot size of 50 *μ*m (rms). Such initial electron pulse parameters can be obtained via single-photon photoemission using the second harmonic from the output of a non-collinear optical parametric amplifier (NOPA).^79–81^ A NOPA provides the frequency tunability necessary to match the work function of various metal candidates such as Ti, stainless steel, Mo, and W. The photocathode is held at a potential *V* = −300 kV with respect to ground. The cathode-anode separation distance along the propagation axis, *d_z_* ≅ 5 cm, confers an average on-axis electric field $〈E_{z}〉=-\frac{\Delta V}{d_{z}}\;≅-6\;\text{MV}/m$. Equipotential lines, calculated using Poisson Superfish,^82^ are shown in panel A of Fig. 2 (red traces). A large increase in the magnitude of the on-axis electric field $\left|E_{z}\right|$ can be observed as we approach the cathode head reaching a maximum value of  50 MV/m at the surface, see Fig. 2(b).

One of the major concerns of the current design is vacuum breakdown that can compromise the stability of our electron source. Critical surface vacuum breakdown fields, $E_{s,c}$, have been measured and found to be $E_{s,c}≅6.5-10\;\text{GV}/m$ for refractory metals.^83–85^ Such critical threshold is commonly expressed as $\left|E_{s,c}\right|=\beta_{m\;}\beta_{g}\left|\Delta V_{c}\right|/d$, where $\beta_{m\;}$ and $\beta_{g}$ are microscopic and geometrical field enhancement factors, respectively, and $\Delta V_{c}$ is the critical applied potential drop over a given separation distance, *d*, between two electrodes. Thus, $\left|\Delta V_{c}\right|/d$ equals the magnitude of the average critical applied field $\left|〈E_{c}〉\right|$, and $\beta_{g}\left|〈E_{c}〉\right|$ becomes what we refer to as the “geometrical critical surface electric field” $\left|E_{g,c}\right|$, which therefore results in $\left|E_{s,c}\right|=\beta_{m\;}\left|E_{g,c}\right|$. Typical values of $\beta_{m\;}$ for polished surfaces lie in the range of 100–300 (with 100 corresponding to mirror-like surface finishing^85^). On the other hand, the magnitude of the maximum geometrical surface electric field $\left|E_{\mathrm{g,max}}\right|$ in previous compact electron gun designs was calculated to be about 20 MV/m and therefore satisfies the condition $\beta_{m}\left|E_{g}\right|<\left|E_{s,c}\right|$. Note that we cannot modify $\left|E_{s,c}\right|$ but $\beta_{m}$ and $\left|E_{g}\right|$ within certain limits with $\beta_{m}\to 1$ for a roughness free surface. Surface conditioning is known to reduce dark current and greatly increase $\left|〈E_{c}〉\right|$^86–88^ by bringing $\beta_{m\;}$ from several hundreds to about 20–50 for refractory metals.^84^ The use of a solid cathode head made of Ti, for instance, is therefore essential to allow for proper surface processing. This is difficult to achieve in back illuminated electron guns due to the implementation of ultrathin film photocathodes that can be easily damaged by arcing. On this subject, the cathode shape was optimized to obtain a maximum geometrical surface electric field $\left|E_{g,max}\right|<60\;\text{MV}/m$ in order to maintain $\beta_{m}\left|E_{g}\right|$ below $\left|E_{s,c}\right|$. In addition, the source design ensures low surface electric fields at the anode electrode ($\left|E_{a}\right|≲5\;\text{MV}/m$), a fact that will greatly mitigate anode-initiated vacuum breakdown.^88^

**FIG. 3.**
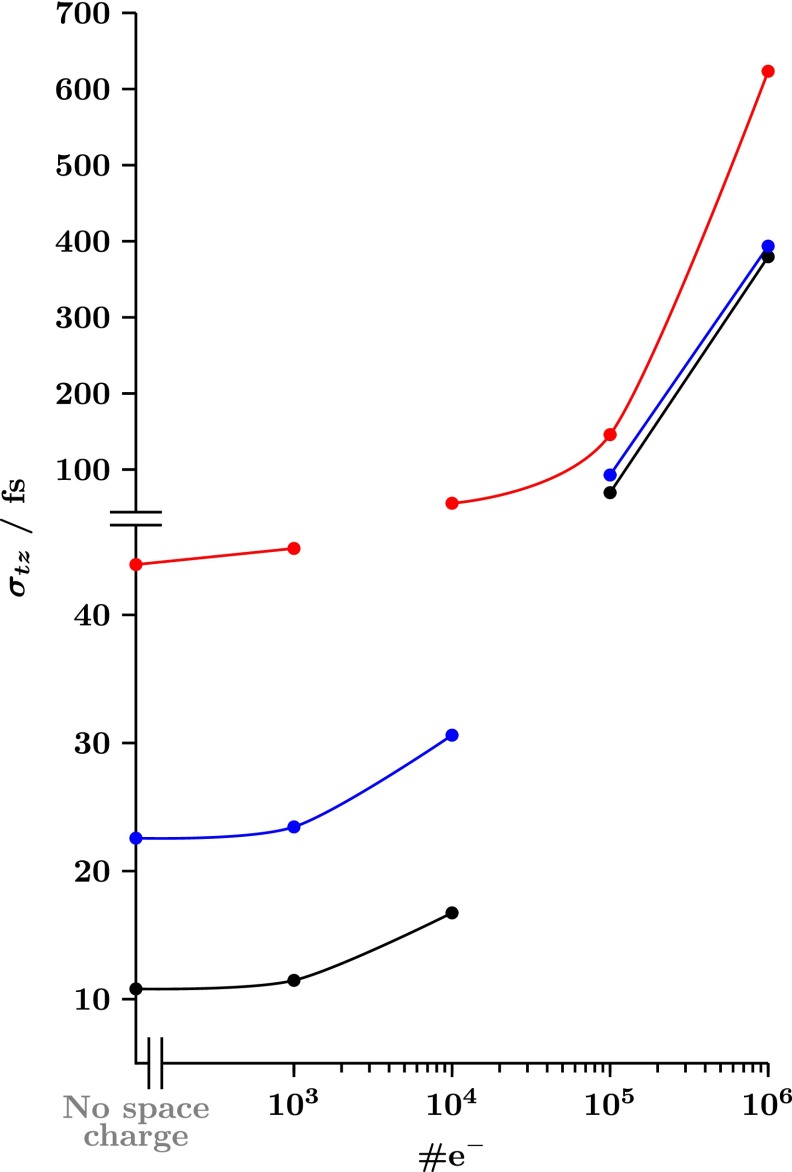
Standard deviation or root-mean-square (rms) electron pulse duration ($\sigma_{tz}$, in fs) as a function of the number of electrons per bunch (#e^−^) obtained from ASTRA particle tracer simulations.^89^ Black trace corresponds to our 300 kV FED design and *d_T_* = 10 cm. Blue and red traces correspond to compact 100 kV FED setups (i.e., large flat cathodes) with *d_T_* = 2 cm and constant electrostatic fields of *E_z_* =−20 MV and −10 MV/m, respectively.

Fig. 3 shows the electron pulse duration $\sigma_{tz}$ (rms) obtained from ASTRA simulations^89^ for different electron source geometries as a function of the number of electrons per bunch. Black trace corresponds to our 300 kV FED electron gun concept for a total electron propagation distance *d_T_* = 10 cm. Blue and red traces refer to the results obtained for conventional 100 kV compact FED setups with flat parallel electrodes, *d_T_* = 2 cm, and constant on-axis electric fields of $E_{z}$ = −20 MV/m and −10 MV/m, respectively. Note that despite the relatively long propagation distance, the proposed design provides $\sigma_{tz}$ < 20 fs for bunches containing 10^4^ electrons, and only $\sigma_{tz}≅\;$12 fs in the limit of low space charge effects. It should be mentioned, however, that the main disadvantage of the proposed electron source is its relatively larger spot size, ≅190 *μ*m (rms), when compared with that of a compact FED setup, ≅55 *μ*m (rms), with the same initial electron beam parameters.

**FIG. 4.**
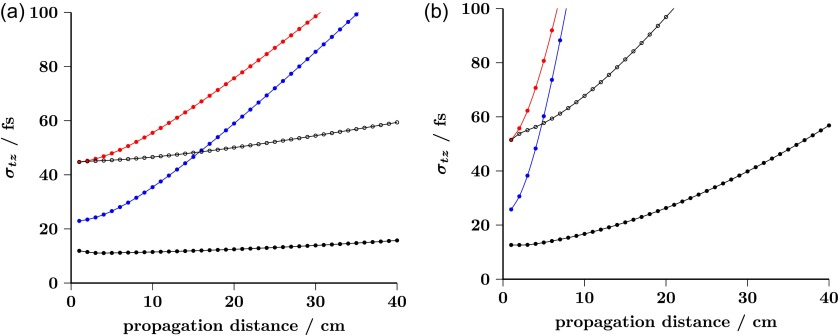
Electron pulse duration $\sigma_{tz}$ (rms) as a function of propagation distance from the cathode surface for bunches containing 10^3^ and 10^4^ electrons, panels (a) and (b), respectively. Black closed dots: our 300 kV FED design. Blue and red dots: compact 100 kV FED setups with constant electric fields of *E_z_* = −20 MV/m and *E_z_* = −10 MV/m, respectively. Black open dots: compact 300 kV FED setup with constant electric field *E_z_* = −10 MV/m.

The most noteworthy feature of this new source design is its ability for delivering ultrashort multi-electron bursts after significantly long propagation distances. As can be seen in Fig. 4(a) by direct comparison against conventional FED setups, geometrical field enhancement plays a key role in minimizing the temporal broadening caused by energy spread at photoemission. Temporal broadening is known to be dominant at the initial stage of electron propagation^90^ and found to be, approximately, inversely proportional to the geometrical surface electric field at the electron birth. Hence, geometrical surface field enhancement appears as a simple means to reduce the temporal broadening introduced by the initial momentum spread.

Therefore, the proposed electrostatic electron source approach lessens the need of using microwave cavities to compensate for such temporal spreading.^67^ Moreover, we can also observe in Fig. 4 an important decrease of the electron pulse expansion rates when *KE* is increased from 100 keV to 300 keV (red and blue dots versus closed and open black dots). This is a consequence of relativistic effects that diminish space-charge repulsive forces by a factor of $\gamma^{-3}$.^91^ We find remarkable (see black closed dots in Fig. 4(b)) that $\sigma_{tz}$ is below 60 fs even for a pulse containing 10^4^ electrons and after 40 cm of propagation. Longer propagation distances may be advantageous for improving transverse electron beam properties owing to the use of additional electron optics and apertures between the electron source and the sample.

Given the extremely short length of the produced electron bursts, we decided to explore the effect of instabilities of the power source on the instrument response time. Fluctuations of the electron gun voltage, $\sigma_{\Delta V}$, result in variations of the arrival time (*t*_0_) of each electron pulse to the sample (or time zero jitter, $\sigma_{t_{0}}$). We found for our 300 kV FED design $\sigma_{t_{0}}/fs\;≅\;4\times 10^{-2}\cdot \sigma_{\Delta V}/\text{eV}\;\cdot d_{T}/\text{cm}$. Hence, voltage drifts of 10 ppm ($\sigma_{\Delta V}=$ 3 eV) and *d_T_* = 10 cm translate into $\sigma_{t_{0}}≅$ 1.2 fs. A state-of-the-art high-voltage power supply is therefore necessary to guarantee that the overall temporal instrument response is not limited by fluctuations of the voltage source.^92^

## CONCLUSIONS

We have presented an electron source that builds solely on electrostatic fields and that is capable of generating ultrashort and bright multi-electron pulses with minimal temporal degradation over long propagation distances. The main two ingredients of this electron source design are: (i) geometrical field enhancement that increases the strength of the electric field at the electron birth and therefore reduces the temporal broadening caused by initial energy spread; and (ii) higher *KE* that helps to diminish the detrimental effects of space charge and leads to a decrease of the electron pulse expansion rate. With a time resolution down to ≅  12 fs (rms), FED instruments based on this new electron gun concept hold great promise in resolving even high-frequency vibrational modes without the necessity of implementing RF-electron pulse rebunching or all-optical electron pulse compression schemes.
